# Oxidative stress as a catalyst in prostate cancer progression: unraveling molecular mechanisms and exploring therapeutic interventions

**DOI:** 10.1007/s12672-025-02245-4

**Published:** 2025-04-03

**Authors:** Yawen Song, Zheng Hou, Longting Zhu, Yan Chen, Jingyu Li

**Affiliations:** 1 Department of Urology, Xijing Hospital of Air Force Military Medical University, Xi’an, China; 2 Department of Urology, Dandong Central Hospital, 70 Renmin Road, Zhenxing District, Dandong, 118000 Liaoning China; 3 First Affiliated Hospital, Army Medical University, Chongqing, China; 4 Department of treatment disease, traditional Chinese medicine, Shizuishan, China

**Keywords:** Prostate cancer, ROS, Antioxidants

## Abstract

Prostate cancer is the second most common malignancy among men worldwide, with its incidence and mortality rates steadily increasing. Although androgen deprivation therapy (ADT) combined with androgen receptor inhibitors has shown significant efficacy in treating prostate cancer, resistance to treatment remains a major challenge, particularly in patients with metastatic prostate cancer. Reactive oxygen species (ROS), a class of highly reactive molecules, can induce oxidative stress within cells, thereby affecting cellular survival and function. In cancer cells, elevated ROS levels not only promote proliferation and invasion but also contribute to the malignancy of tumors by modulating the tumor microenvironment, enhancing angiogenesis, and facilitating extracellular matrix remodeling. This review systematically explores the pathways of ROS generation in prostate cancer, their interaction with the androgen receptor signaling pathway, and the role of external factors such as obesity and aging in promoting ROS production. The findings highlight that ROS drive prostate cancer progression through multiple mechanisms, including altering the tumor microenvironment, activating the unfolded protein response (UPR), and regulating miRNA expression. By providing a comprehensive analysis of ROS-mediated mechanisms in prostate cancer, this review offers new insights into the development of targeted antioxidant therapeutic strategies.

## Introduction

Prostate cancer (PCa) is the second most common malignancy among men, with a significant global impact. According to the 2022 report from the International Agency for Research on Cancer, there were 1.467 million new cases of PCa in 2022, accounting for 14.2% of all male cancers, second only to lung cancer. The disease also resulted in 397,000 deaths, ranking fifth in cancer-related mortality among men [1]. PCa typically begins as intraepithelial neoplasia and progresses to androgen-dependent adenocarcinoma, eventually leading to castration-resistant PCa (CRPC) as the tumor advances [2]. Given that prostate tumor growth is driven by androgens and androgen receptors (AR), standard clinical treatment involves androgen deprivation therapy (ADT) combined with androgen receptor inhibitors, often supplemented with radiotherapy or chemotherapy [3]. While ADT has shown significant efficacy, a subset of patients with metastatic PCa eventually develop resistance to these therapies, which can lead to fatal outcomes [4].

Reactive oxygen species (ROS), a group of highly reactive molecules containing oxygen, including superoxide anion (O_2_^−^), hydrogen peroxide (H_2_O_2_), singlet oxygen, and hydroxyl radicals (·OH), can interact with lipids, proteins, and nucleic acids within cells, leading to oxidative stress [5]. In normal cells, an imbalance in the redox environment leading to increased ROS typically triggers cell death signals [6]. However, in cancer cells, elevated ROS levels are often associated with enhanced proliferation and drug resistance [7]. In the tumor microenvironment, ROS not only contribute to immune evasion but also modulate the microenvironment to promote tumor growth [8]. Studies have shown that increased ROS levels are linked to a higher risk of developing PCa [9]. Furthermore, PCa cell growth has been associated with androgen-mediated increases in ROS and subsequent autophagy enhancement [10].

Understanding how ROS drive PCa progression is therefore crucial. This review provides a comprehensive overview of ROS production in PCa, the interplay between androgens and ROS, the role of external factors such as obesity and aging in promoting ROS generation, and how elevated ROS levels alter the PCa microenvironment, promoting inflammation, activating the unfolded protein response (UPR), and regulating miRNA expression, all of which contribute to the progression of PCa.

## Origins of oxidative stress in prostate cancer

**Fig. 1:**
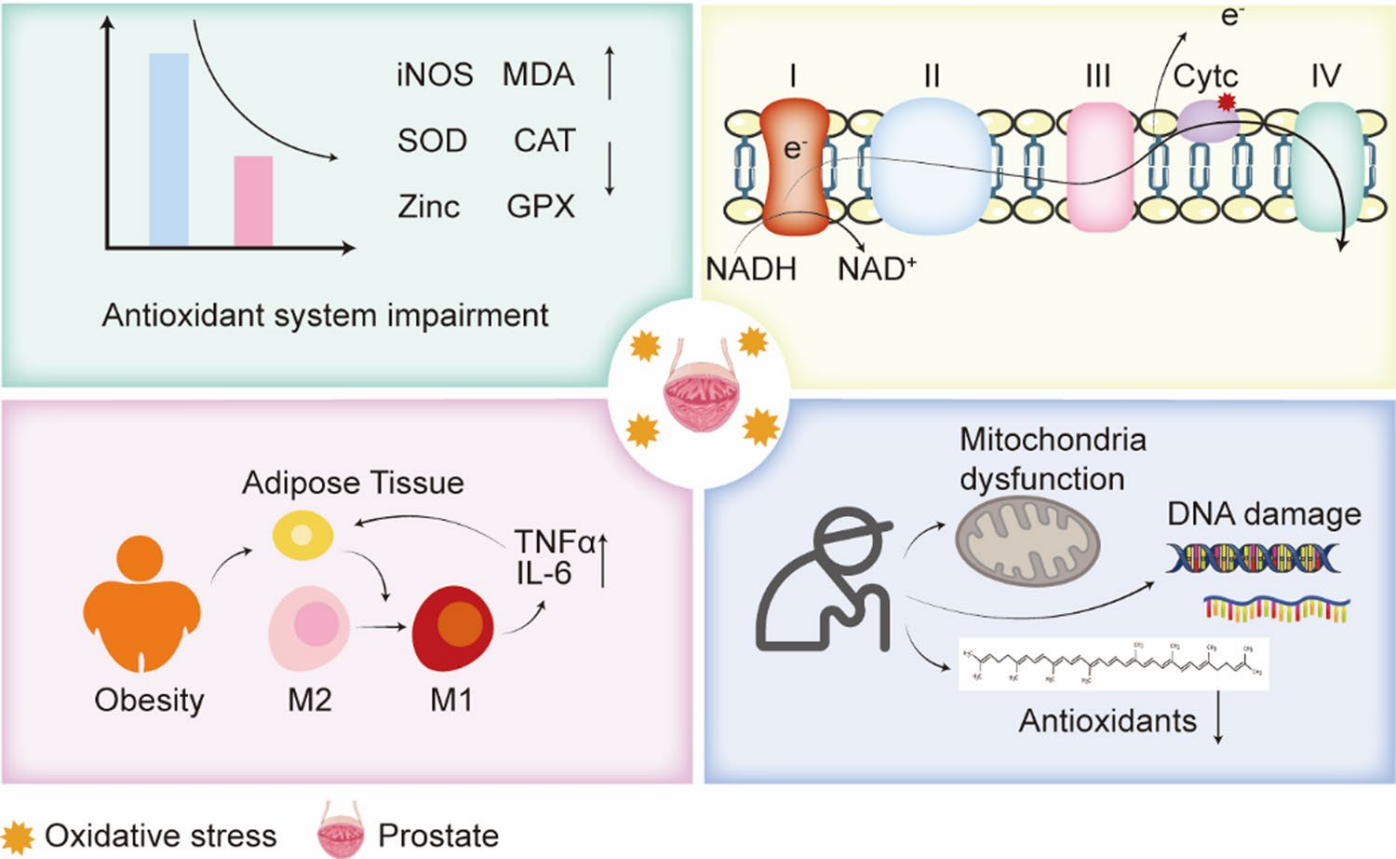
Sources of Reactive Oxygen Species in Prostate Cancer. In PCa, the antioxidant defense system is compromised, with a reduction in antioxidant enzymes such as SOD, CAT, and GPX. Androgens further exacerbate this condition by disrupting the ETC, leading to electron leakage and subsequent ROS production. Exogenous factors like obesity and aging contribute to increased production of inflammatory cytokines, mitochondrial damage, DNA damage, and a decline in antioxidants, all of which collectively enhance ROS generation

Research indicates that each cell undergoes approximately 1.5 × 10^5^ oxidative assaults daily [11]. In the context of PCa, there is substantial evidence that oxidative systems are compromised, leading to an exacerbation of ROS production [12]. When ROS levels increase or the cellular capacity to mitigate oxidative stress diminishes, the frequency of oxidative assaults on cells rises, ultimately resulting in heightened oxidative stress. The sources of oxidative stress in the prostate are illustrated in Fig. 1.

### Dysregulation of antioxidant defense mechanisms in prostate cancer

It has been well established that oxidative stress is elevated in cancer cells, and in PCa, one of the primary sources of this heightened oxidative stress is the impairment of the antioxidant defense system. This impairment includes a reduction in the levels of various enzymes and antioxidants, as well as an increase in oxidant levels [13]. Oxidative stress arises from both reactive nitrogen species (RNS) and ROS. Research by Baltaci et al. demonstrated a significant upregulation of inducible nitric oxide synthase (iNOS), which produces nitric oxide (NO), in both PCa and benign prostatic hyperplasia (BPH) samples, with higher expression observed in PCa. This suggests that NO produced by iNOS may contribute to the progression of PCa, although the precise mechanisms require further elucidation [14]. Further studies by Arsova et al., involving 107 PCa patients, revealed that compared to the general population, PCa patients exhibited significantly decreased serum activities of CuZn-SOD, catalase (CAT), and glutathione peroxidase (GPX), alongside increased levels of malondialdehyde (MDA) [15]. Elevated MDA levels, a byproduct of lipid peroxidation, indicate an increase in lipid peroxidation in PCa patients, corroborating findings by Yossepowitch et al., which reported increased sensitivity to lipid peroxidation in the serum of PCa patients [12]. Similarly, research by Yilmaz et al. also demonstrated elevated plasma MDA levels and reduced antioxidant activity in PCa patients [16]. Glutathione peroxidase 3 (GPx3), an important component of the cellular antioxidant defense system, catalyzes the reduction of hydrogen peroxide, organic hydroperoxides, or lipid peroxides to their corresponding alcohols. High-fat diets have been shown to reduce the expression of GPx3 in the prostate tissue of mice, and cholesterol treatment of PC-3 cells resulted in reduced GPx3 levels, increased H_2_O_2_ levels, and enhanced proliferation of PC-3 cells [17]. This suggests that diminished antioxidant capacity may be one of the mechanisms through which obesity promotes PCa.

In addition to direct impacts on antioxidant enzyme expression, PCa can also affect enzyme function through post-translational modifications. Chaiswing et al. observed a significant increase in the expression of manganese superoxide dismutase (MnSOD) and thioredoxin 1 (TRX1) in PCa tissues, but MnSOD activity increased only slightly (enzyme activity increased 0.5-fold, while protein expression increased twofold), and TRX1 activity remained unchanged. Further analysis revealed that these enzymes underwent post-translational modifications, where the oxidation of cysteine residues at active sites led to reduced enzyme activity. Additionally, the study reported that both extracellular SOD protein and enzyme activity, as well as the levels of reduced TRX1, were markedly decreased in PCa tissues compared to normal tissues [18]. This reduction in SOD activity and reduced TRX1 levels contributes to the imbalance of the antioxidant system within the tumor microenvironment, exacerbating oxidative stress.

In addition to the impairment of the antioxidant enzyme system, PCa tissues also exhibit a reduction in antioxidants. Zinc levels progressively decrease from normal tissue to BPH to PCa tissues, indicating the critical role of zinc ions in maintaining normal prostate function [19]. Zinc ions have been reported to inhibit cell cycle progression and apoptosis, thereby preventing the development of PCa [16]. Supplementation with low doses of zinc has been shown to effectively reduce both the lethality risk and all-cause mortality associated with PCa [20]. Moreover, zinc ions have been reported to lower lipid peroxidation levels [21], which may explain the elevated lipid peroxidation observed in PCa patients. As zinc is a necessary cofactor for SOD, its decreased levels could potentially account for the reduced SOD activity observed in PCa tissues. Beyond zinc ions, Almushatat's study demonstrated a significant reduction in circulating levels of lutein, lycopene, β-carotene, and α-tocopherol in the blood of PCa patients, with lutein and lycopene levels showing a significant negative correlation with MDA levels. This suggests a marked decrease in antioxidant levels in the blood of PCa patients, which more accurately reflects the progression of the disease than systemic inflammation [22]. This finding is consistent with the report by Maramag et al. that vitamin C can inhibit PCa [23]. Glutathione (GSH), another crucial antioxidant, works through its thiol group to reduce oxidized components into stable metabolites under the action of glutathione S-transferase. Both PCa and BPH patients have been observed to have reduced GSH levels and increased glutathione S-transferase activity, with GSH levels being lower in PCa compared to BPH patients. This indicates that PCa cells require higher levels of GSH to counteract the increased oxidative metabolites present in the blood.

### Androgen-induced ROS augmentation and its oncogenic implications

The proliferation of PCa is heavily dependent on androgens, which exert their effects by binding to AR. In PCa tissues, AR expression is significantly upregulated [24]. The increase in ROS can further activate the AR pathway, thereby promoting the progression of PCa [25]. Studies have shown that in androgen-responsive PCa cells, androgen exposure leads to a decrease in GSH levels and an increase in γ-glutamyl transpeptidase activity, both of which can be counteracted by antioxidant treatment. Further research suggests that this oxidative state may be due to androgens enhancing mitochondrial activity, as evidenced by increased oxygen consumption rates and catalase activity, leading to elevated ROS production [26]. Mitochondria, as the primary energy-producing organelles in cells, generate ATP through the mitochondrial electron transport chain (ETC), during which electron leakage occurs, contributing to ROS formation [27]. Electron leakage primarily takes place at Complex I and Complex III of the ETC, with cytochrome c playing a key role in electron transfer at Complex III [28]. Studies have demonstrated that androgen-treated PCa cells upregulate the expression of p66Shc, which, under the influence of androgens, translocates to mitochondria and interacts with cytochrome c, exacerbating electron leakage and thereby increasing ROS production and promoting PCa cell proliferation [29]. Additionally, p66Shc can competitively bind to the C-SH3 domain of grb2 via its N-terminal CH2 domain, leading to the dissociation of sos1 from grb2 and the formation of a trimeric complex with eps8 and e3b1. This trimeric complex enhances RAC1 activity, further triggering oxidative stress responses [30].

NADPH oxidase, another major source of ROS, generates superoxide by transferring electrons from NADPH to molecular oxygen, thereby facilitating the production of other ROS types. NADPH oxidase exists in multiple isoforms, including Nox1, Nox2, Nox4, and Nox5 [31]. Different types of PCa cells exhibit varying levels of Nox expression. For instance, Höll et al. found that Nox5 expression is elevated in LNCaP and PC-3 cell lines but not in the androgen-independent DU145 cells [32]. Research in neuronal cells has shown that androgens can activate NADPH oxidase through AR, leading to increased ROS production [33]. This suggests that androgens may contribute to Nox-mediated ROS upregulation, explaining why Nox5 upregulation is absent in androgen-independent DU145 cells. Furthermore, Höll et al. observed discrepancies between in vitro and in vivo results, with no significant upregulation of Nox5 in PCa tissues in vivo. The authors hypothesize that Nox5 activation may be dependent on post-translational modifications [32].

CRPC is a type of tumor that continues to grow despite androgen levels being reduced to castrate levels. Studies have shown that compared to androgen-responsive tumors, CRPC can downregulate SOD2 expression, which contributes to the accumulation of ROS while simultaneously promoting the expression of androgen-responsive genes such as *VEGFA* and *FKBP5*. This enhancement of AR-DNA binding activity can be counteracted by antioxidants [34]. This indicates that SOD2 deficiency-induced ROS upregulation and AR activation play critical roles in CRPC progression.

These studies collectively suggest that the increase in ROS-induced oxidative stress can activate AR, promoting PCa development. Concurrently, the activated AR pathway can further increase ROS production, leading to a vicious cycle of oxidative stress and malignant proliferation in PCa cells.

### Exogenous influences on oxidative stress and prostate cancer pathogenesis

Studies have shown that the majority of PCa patients are diagnosed after the age of 65, with the risk of developing PCa significantly increasing in older individuals [35]. Age-related changes in prostaglandin levels are likely linked to oxidative stress [36]. As patients age, there is a marked increase in the proportion of individuals with higher Cancer of the Prostate Risk Assessment scores, indicating that older patients are more susceptible to high-risk PCa [37]. Additionally, survival rates for PCa patients significantly vary with age; for instance, the mortality rate for patients diagnosed before the age of 70 is 17%, but this increases to 21% for those diagnosed after 70 [38]. Furthermore, PCa mortality rates continue to rise with age, peaking at 85 years and older [38]. This suggests a strong association between age and PCa, likely driven by increased ROS levels. ROS can damage cellular structures and tissues, leading to functional decline, which is a hallmark of aging [39]. Aging cells often experience mitochondrial dysfunction and DNA damage, resulting in heightened sensitivity to oxidative stress [40], consistent with findings by Szewczyk et al., who reported a significant decline in GSH levels in red blood cells of elderly patients with PCa or BPH, leading to reduced antioxidant system performance [41]. Research by Peng et al. found that the concentration of the antioxidant lycopene is inversely related to age, with plasma lycopene levels significantly decreasing as age increases [42]. Furthermore, lycopene supplementation has been shown to effectively reduce the risk of developing PCa [43], further confirming the protective role of antioxidants in mitigating age-related oxidative stress and preventing PCa.

In addition to aging, a high-fat diet and obesity are also associated with PCa [44]. High-fat diets can lead to an increase in adipose tissue and inflammation. Persistent inflammation can promote the conversion of anti-inflammatory M2 macrophages to a pro-inflammatory state, resulting in the further release of inflammatory cytokines such as IL-6 and TNFα [45]. These cytokines stimulate adipocytes to produce ROS, leading to elevated oxidative stress, which in turn promotes further cytokine release from macrophages [46]. This positive feedback loop results in sustained oxidative stress and damage to adipocytes. The released inflammatory cytokines IL-6 and TNFα have been reported as tumor-promoting factors that play significant roles in the initiation, progression, and prognosis of PCa [47]. Additionally, this feedback loop further reduces the expression of antioxidant enzymes, exacerbating the imbalance in oxidative stress [48]. Elevated ROS levels can activate the JNK pathway and inhibit the phosphorylation of insulin receptor substrate 1, leading to insulin resistance [49]. Insulin resistance causes hyperinsulinemia, which, by reducing insulin-like growth factor-binding proteins, results in elevated levels of IGF-I [50]. Increased IGF-I levels further raise free estrogen levels [51]. Research has shown that polymorphisms in estrogen-related genes are closely associated with the risk of developing PCa [52]. Hormonal imbalances due to declining androgen levels and unchanged estrogen levels with age are considered potential risk factors for PCa [53]. Furthermore, estrogen can increase the risk of PCa by upregulating estrogen receptors [54]. Bosland et al. demonstrated that while estrogen alone causes chemical castration in rats, the combination of estrogen and androgen increased the incidence of PCa in NBL rats from 35% with testosterone alone to nearly 100% [55]. This further confirms estrogen's role in enhancing androgen-induced PCa. These findings suggest that obesity not only increases ROS production through the release of inflammatory cytokines but also promotes PCa development by disrupting hormonal balance.

### The role of oxidative stress in prostate cancer progression and prognosis

Compared to normal cells, PCa cells are capable of synthesizing more antioxidants, such as HO-1, Nrf2, and GPXs, to cope with the continuous production of ROS [56]. As a second messenger within the cell, ROS has been shown to regulate processes such as cell proliferation and differentiation. Kumar et al. demonstrated that, compared to normal epithelial cells, prostate cancer cells produce higher levels of ROS, with PC3 cells generating more ROS than DU145 and LNCaP cells. This suggests that high levels of ROS production are associated with increased invasiveness and malignancy in cancer cells [57]. Moreover, the use of the Nox inhibitor diphenyleneiodonium significantly suppressed the proliferation of prostate cancer cells [57], indicating that ROS is closely linked to cancer cell invasion and proliferation at the cellular level. Biesiadecki et al. assessed the levels of oxidative stress markers in the serum and urine of 50 prostate cancer patients and found that as the cancer stage advanced, there was a further decrease in serum thiol groups and TAC, along with an increase in lipid peroxidation products like MDA, suggesting that oxidative damage worsens as the cancer progresses [58]. Beyaztas et al. also reported that oxidative stress and inflammation levels were significantly elevated in prostate cancer patients compared to healthy individuals [59]. Furthermore, studies have shown that ROS can promote the progression of prostate inflammation through the NLRP3 pathway [60]. C-reactive protein (CRP), a hallmark of inflammation, has been investigated as a prognostic marker in prostate cancer. Thurner et al. evaluated the prognostic significance of elevated plasma CRP levels in 261 prostate cancer patients undergoing radiotherapy. Their study revealed that elevated plasma CRP levels (≥ 8.6 mg/L) were associated with poorer cancer-specific survival, overall survival, and disease-free survival in prostate cancer patients. Importantly, this association was independent of other prognostic indicators, such as tumor stage, Gleason score, and prostate-specific antigen levels at diagnosis [61]. These findings collectively indicate that oxidative stress plays a pivotal role in the onset and progression of prostate cancer and is strongly associated with poor prognosis.

## Mechanistic pathways of ROS in prostate cancer advancement

### ROS-mediated modulation of the tumor microenvironment in prostate cancer

The tumor microenvironment, which comprises tumor cells, non-tumor cells, tumor vasculature, extracellular matrix (ECM), biochemical factors, and mechanical environment [62], has been shown to significantly influence tumor growth, invasion, and progression.

Cancer cells, due to their excessive proliferation, require increased nutrient supply, which often results in enhanced angiogenesis [63]. For example, vascular endothelial growth factor (VEGF) is reported to be highly expressed in PCa patients compared to healthy individuals, with further upregulation observed in metastatic PCa cases [64]. ROS have been identified as key factors in promoting angiogenesis in cancer cells [65]. The pathways through which ROS enhance angiogenesis include the PI3K/AKT/mTORC1, MAPK/ERK, and IKK/NF-κB pathways [66, 67]. Elevated ROS levels can activate the catalytic subunit p110 of PI3K, which subsequently phosphorylates PIP2 to generate PIP3. PIP3 recruits AKT, which, through the actions of PDK1 and mTORC2, becomes phosphorylated and activated. Activated AKT further stimulates mTORC1, leading to increased expression of hypoxia-inducible factor 1-alpha (HIF-1α), which directly promotes VEGF-mediated angiogenesis [68]. Additionally, ROS can activate Ras proteins, which, as GTPase switch proteins, activate Raf kinase and initiate a MAPK cascade, activating downstream kinases MEK and ERK. Activated ERK translocates to the nucleus, upregulating the transcription of HIF-1α and VEGF, further promoting angiogenesis [69]. IκB proteins, which inhibit NF-κB by sequestering it in the cytoplasm, can be phosphorylated by IκB kinase (IKK) activated by ROS, leading to the release and nuclear translocation of NF-κB. NF-κB then upregulates VEGF and pro-inflammatory cytokines such as TNF-α and IL-6, further driving angiogenesis [70].

In addition to promoting angiogenesis, ROS have also been reported to activate matrix metalloproteinases (MMPs). MMPs degrade ECM components, including collagen, elastin, and glycosaminoglycans, thereby facilitating tumor invasion and metastasis by breaching physical barriers [71, 72]. The activation of MMPs is closely associated with oxidative stress, and ROS have been shown to promote MMP expression through the PI3K/AKT and IKK/NF-κB pathways [73]. In PCa, upregulation of MMP expression not only leads to ECM remodeling and reduced stiffness but also promotes tumor migration [74, 75]. Zhou et al. demonstrated that mice lacking membrane-type matrix metalloproteinase I exhibit significant angiogenesis defects, suggesting that MMPs also play a role in promoting angiogenesis, thereby aiding tumor invasion [76, 77].

Within the cellular microenvironment, fibroblasts are key components of the ECM, synthesizing and secreting collagen and other ECM components to maintain tissue structure and elasticity. In the cancer microenvironment, fibroblasts are activated into cancer-associated fibroblasts (CAFs). CAFs overproduce ECM components and secrete lysyl oxidase, promoting collagen fiber crosslinking and increasing matrix rigidity, a characteristic feature of PCa [78]. A high proportion of CAFs in PCa tissue is also associated with increased epithelial-mesenchymal transition (EMT) [79, 80]. A significant number of CAFs identified in invasive adenocarcinomas express α-smooth muscle actin (α-SMA), a type of fibroblast known as myofibroblasts [62]. ROS have been shown to be crucial in driving the conversion of fibroblasts into myofibroblasts. Mitochondrial ROS promote the activation of transforming growth factor-beta (TGF-β), which, in PCa tissue, stimulates NOX4-mediated ROS production and JNK phosphorylation, leading to the conversion of fibroblasts into myofibroblasts. This conversion can be inhibited by targeting mitochondrial ROS with antioxidants or selenium, further highlighting the importance of ROS in fibroblast-to-myofibroblast conversion [81, 82]. Activation of TGF-β has also been shown to induce a phenotypic switch in fibroblasts, converting them into SPP1 + myofibroblast-type CAFs that contribute to resistance to androgen deprivation therapy (ADT) and the progression of CRPC [83]. Moreover, ROS can increase the accumulation of HIF-1α and chemokine CXCL12, further promoting the conversion of fibroblasts into myofibroblasts, a process that can be inhibited by antioxidant use [84].

Antognelli et al. demonstrated that the accumulation of MG-H1 in prostate cancer cells can downregulate the expression of PD-L1, leading to PD-L1-mediated suppression of cytotoxic CD^8+^ T cells and promoting immune escape of the tumor [85]. It is known that MG-H1 can induce the generation of ROS and cause oxidative stress [86], suggesting that ROS may play a role in MG-H1-mediated tumor immune escape. The "seed and soil" hypothesis suggests that tumors require a favorable microenvironment for growth and expansion, which supports their metastatic potential [87]. MG-H1 has been reported to promote ROS production and activate the NF-κB signaling pathway through interaction with the receptor RAGE, thereby facilitating the dedifferentiation and increased mineralization activity of osteoblasts, thus creating a microenvironment conducive to osteoblastic metastasis of prostate cancer cells [88]. The generated ROS can further activate the NF-κB pathway [89], which in turn enhances ROS production [90], forming a positive feedback loop that further promotes osteoblast dedifferentiation, creating a favorable growth environment for prostate cancer bone metastasis. These studies highlight the crucial role of ROS in MG-H1-mediated prostate cancer immune escape and bone metastasis.

### Synergistic interactions between ROS and inflammatory processes in prostate cancer

Chronic inflammation is a common pathological feature in PCa tissues. Research by Nickel et al. has demonstrated a correlation between chronic prostatitis and the severity of lower urinary tract symptoms, with this correlation increasing as inflammation progresses [91]. This suggests that inflammation may play a role in the progression of PCa. Inflammation not only leads to cellular and genomic damage but also alters the tumor microenvironment, enhancing cell proliferation, promoting angiogenesis, and facilitating tissue repair—factors that collectively contribute to tumor growth [92]. The glutathione S-transferase P1 (GSTP1) gene plays a critical role in the transition from inflammation to PCa. Proliferative inflammatory atrophy (PIA), often observed in early PCa, has been associated with significant hypermethylation of the CpG island in the GSTP1 promoter region [93]. This hypermethylation has also been observed in PCa cells [94]. GSTP1, a member of the GST family, catalyzes the reaction between GSH and ROS, thereby mitigating oxidative stress within cells. Hypermethylation, a common epigenetic modification in cancer cells, leads to gene silencing, which impairs the ability of prostate cells to clear ROS effectively, resulting in increased oxidative stress [95]. Conversely, Parsons found that normal peripheral zone epithelial cells rarely express GSTA1, whereas GSTA1 expression is significantly elevated in PIA but reduced in adenocarcinoma [96]. The high expression of GSTA1 in PIA suggests that the affected cells are experiencing increased oxidative stress, while the low expression of GSTA1 in adenocarcinoma indicates a deficiency in detoxifying ROS, implicating increased oxidative stress in the transition from prostatitis to PCa. The oxidative stress caused by NO and various ROS produced during inflammation can further exacerbate the inflammatory response [97]. This progression of inflammation leads to the upregulation of cytokines such as IL-6 and TNF-α, and induces the expression of cyclooxygenase-2 (COX-2) [98, 99]. COX-2 and downstream enzymes convert arachidonic acid into prostaglandins [100]. Prostaglandins exert their effects through binding to their receptors, and research has shown that prostaglandin receptor 4 is significantly upregulated in CRPC compared to androgen-sensitive PCa, suggesting that prostaglandins may contribute to the development of castration resistance in PCa [101].

### Interconnection between oxidative stress and er stress in prostate cancer

**Fig. 2:**
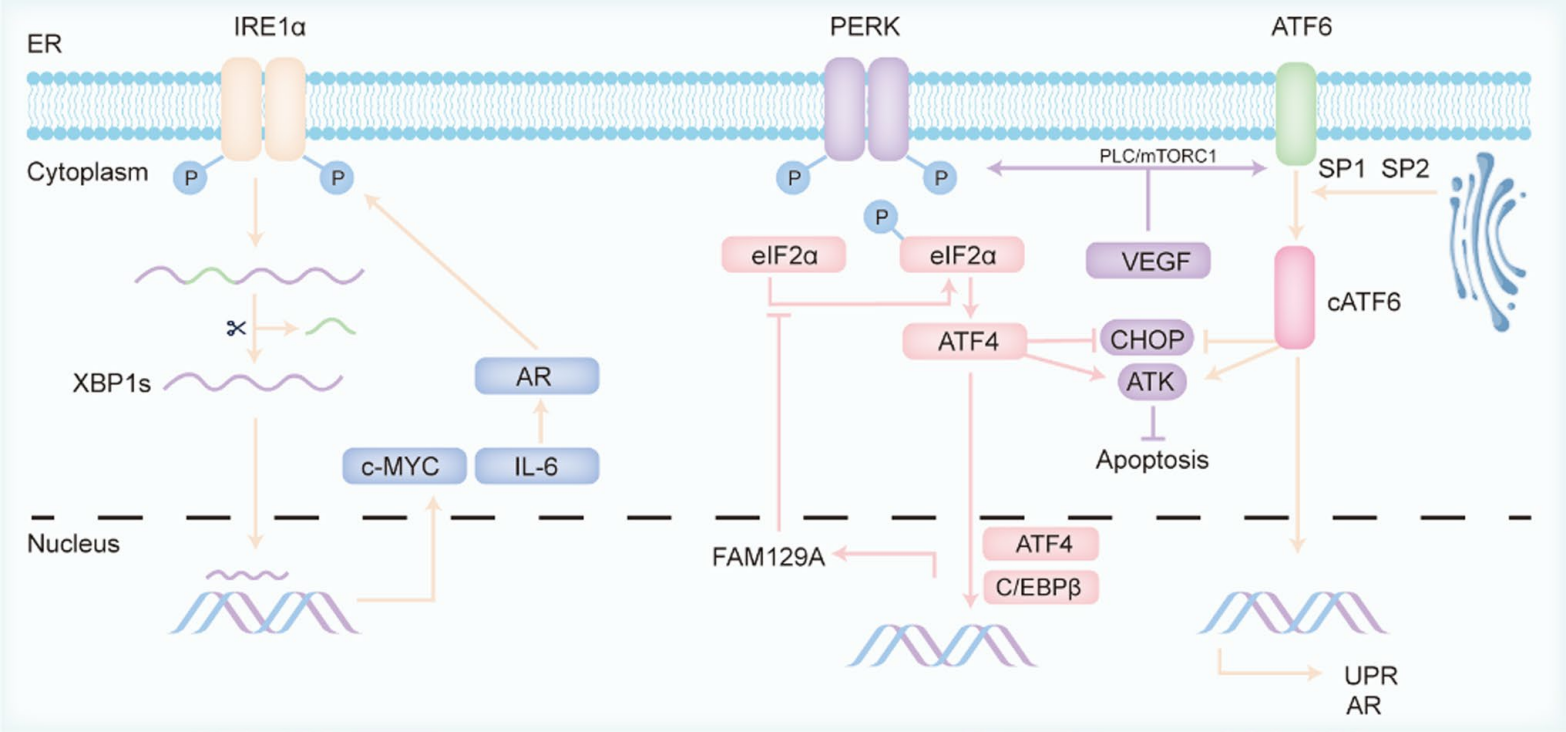
Role of ROS-activated upr in prostate cancer progression. The UPR is activated via the IRE1α, PERK, and ATF6 pathways. IRE1α generates XBP1s, which regulates c-MYC and IL-6, leading to metabolic reprogramming and AR activation. PERK and ATF6 promote PCa progression by inhibiting apoptosis through ATF4 and cATF6 and by activating AR

The endoplasmic reticulum (ER) is a crucial organelle responsible for protein folding within cells. Increased oxidative stress can disrupt the formation of disulfide bonds within the ER, leading to ER stress. When ER stress exceeds the ER's capacity to maintain homeostasis, the unfolded protein response (UPR) is activated. The UPR primarily involves the activation of the IRE1α, PERK, and ATF6 pathways [102], which subsequently trigger downstream signaling cascades (as illustrated in Fig. 2). Given that the prostate's primary function is to produce seminal fluid, requiring extensive protein synthesis, the UPR is closely linked to the progression of PCa [103].

XBP1s, a key effector of the IRE1α pathway, regulates the expression of a series of genes. Sheng's research demonstrated that XBP1s is significantly overexpressed in PCa compared to normal tissues, and inhibiting the IRE1α pathway markedly reduces PCa cell proliferation [104]. This finding is consistent with subsequent studies showing that MKC8866, an IRE1α RNase-specific inhibitor, can effectively halt PCa proliferation [105]. Further transcriptome analysis revealed that the IRE1α/XBP1s pathway is essential for the activation of c-MYC, a key driver of tumor growth, by upregulating its expression [105]. MYC has been found to drive transcriptional reprogramming, enabling PCa cells to become less dependent on AR-regulated gene expression, thereby promoting the transition to CRPC [106]. Additionally, Yang et al. found that overexpression of IRE1α can activate AR through the increased secretion of the pro-inflammatory cytokine IL-6. The activated AR further enhances IRE1α expression, creating a positive feedback loop that promotes PCa proliferation [107]. The increased expression of IL-6, a major pro-inflammatory factor in prostatitis, suggests that inflammation may contribute to UPR-mediated PCa cell proliferation [108].

While research on the PERK and ATF6 pathways in PCa is limited, studies on ER stress in ERG transgenic mice have shown that upregulation of PERK and ATF6 contributes to radioresistance in PCa [109]. During ER stress, the proteases SP1 and SP2 translocate from the Golgi apparatus to the ER, where they cleave and activate ATF6. Activated ATF6 enhances the expression of UPR genes and AR-related genes, promoting PCa progression [110]. As discussed earlier, ROS can upregulate VEGF expression. Recent studies indicate that VEGF can activate PERK and ATF6 through the PLC/mTORC1 pathway [111]. Activated PERK and ATF6 not only further phosphorylate AKT to promote angiogenesis but also downregulate CHOP expression, suppressing apoptosis and thus facilitating PCa proliferation [111]. ATF4, a crucial regulator in the PERK pathway, is activated by the phosphorylation of eIF2α. Once activated, ATF4 translocates to the nucleus and promotes the expression of UPR-related genes [112]. Research has shown that ATF4, in conjunction with C/EBPβ, binds to a response element in the promoter of the gene *FAM129A* (family with sequence similarity 129 member A) and promotes its transcription. FAM129A supports PCa survival by inhibiting cellular senescence and apoptosis [113]. Additionally, FAM129A inhibits the phosphorylation of eIF2α, suggesting that FAM129A acts as a negative feedback regulator that limits UPR activation, thereby reducing cell death [113].

### Regulatory roles of non-coding RNAs in ROS-driven prostate cancer progression

MicroRNAs (miRNAs) are a class of small non-coding RNAs that function by degrading mRNA or inhibiting its translation. Recent studies have shown that miRNAs play a critical role in the development and progression of PCa [114]. Specifically, miR-21 and miR-142 are significantly upregulated in both PCa and CRPC patients compared to those with BPH, with miR-21 expression being even higher in CRPC patients than in those with PCa [115]. This finding is consistent with research by Jajoo et al., which demonstrated that miR-21 expression is significantly higher in highly invasive PC-3 M-MM2 cells compared to non-invasive LNCaP cells, suggesting that miR-21 may enhance the invasive capabilities of PCa tumors [116]. miR-21 promotes tumor growth by downregulating tumor suppressor genes such as PDCD4. The 3' untranslated region of the PDCD4 gene is directly targeted by miR-21, and PDCD4 expression is inhibited in a dose-dependent manner by miR-21 [117]. The suppression of PDCD4 correlates with higher Gleason scores and promotes androgen-independent growth of PCa tumors [117]. Maspin, another tumor suppressor gene, has also been identified as a target of miR-21 [118]. Studies have shown that miR-21-mediated suppression of maspin leads to a loss of self-renewal capacity and induces senescence in PCa tumors under suspension culture conditions. In contrast, PCa tumors that do not express maspin exhibit strong stem cell-like properties and tumorigenicity [119]. Additionally, maspin expression enhances the sensitivity of PCa to salinomycin, induces redifferentiation of PCa tumors, and inhibits bone matrix remodeling and angiogenesis, thereby preventing bone metastasis of PCa [120]. These findings underscore the importance of maspin expression in suppressing PCa tumor progression. Further research suggests that maspin's tumor-suppressive effects may be related to its association with GST. In DU145 cells, maspin activity consistently correlates with GST activity, and the maspin-GST complex inhibits ROS production and reduces VEGF-A expression. When GST is inhibited, maspin loses its ability to regulate ROS, indicating that maspin, as a downstream target of miR-21, plays a role in maintaining intracellular oxidative stress balance [121]. In fact, miR-21 itself is regulated by intracellular oxidative stress, primarily through the involvement of NADPH oxidase. NADPH oxidase has been shown to promote PCa proliferation, and its activity is regulated by ERK1/2. Phosphorylation of the p47(phox) subunit by ERK1/2 activates NADPH oxidase [122]. Studies have demonstrated that siRNA-mediated inhibition of the p47(phox) and p22(phox) subunits of NADPH oxidase not only prevents its activation but also reduces miR-21 expression. Similarly, treatment of PCa cells with the NADPH oxidase inhibitor diphenyleneiodonium or the antioxidant NAC also lowers miR-21 expression levels [116]. In both PC-3 M-MM2 cells and PCa mouse models, the use of PI3 kinase inhibitors reduces AKT phosphorylation and miR-21 levels, indicating that miR-21 expression is dependent on the PI3K/AKT pathway [123]. The PI3K/AKT pathway can be activated by oxidative stress, which may explain the mechanism by which oxidative stress regulates miR-21 expression [124]. Moreover, ROS-activated miR-21 can reduce SOD2 expression, thereby disrupting the antioxidant system. This positive feedback loop further promotes the production of both ROS and miR-21 [125]. IL-6, a key pro-inflammatory cytokine in prostatitis, also plays a role in regulating miR-21 expression. IL-6 and STAT3 can bind to an enhancer upstream of the miR-21 gene, promoting its expression [126]. Given that both IL-6 and STAT3 can be activated by oxidative stress [97, 127], it is likely that they also participate in the regulation of miR-21 by oxidative stress.

### ROS and prostate cancer cell death

PCa cells are more sensitive to fluctuations in ROS levels compared to normal cells. When ROS accumulate excessively within the cell, they may induce secondary oxidative damage, leading to cell death, including apoptosis, autophagic cell death, and ferroptosis [56]. Apoptosis is mediated by two major pathways: the mitochondrial pathway and the death receptor pathway. Mitochondria are not only the primary source of ROS generation but also play a pivotal role in regulating intrinsic apoptosis [128]. ROS can trigger the opening of the mitochondrial permeability transition pore by modulating the Bcl-2 family of proteins, leading to mitochondrial membrane potential loss, which subsequently promotes mitochondrial-dependent apoptosis [129]. The death receptor pathway, on the other hand, mediates apoptosis through the binding of specific pro-apoptotic ligands to their corresponding death receptors. It has been reported that ROS upregulate the expression of death receptors such as TRAIL-R2 and Fas on the membrane of PCa cells, thereby enhancing the transmission of exogenous apoptotic signals [130]. Autophagic cell death, also known as type II programmed cell death, has been shown to be associated with mitochondrial dysfunction. Autophagy, as a tumor-suppressive mechanism, helps alleviate oxidative stress. However, when ROS levels exceed the cell's antioxidant capacity, it can lead to the occurrence of autophagic cell death [131]. Studies have shown that the arsenic compound KML001 can promote the expression of the autophagy-specific protein LC3 through ROS, thus inducing autophagic cell death in PCa cells [132]. Ferroptosis is a unique form of programmed cell death, characterized by the high expression of HO-1 [133]. Excessive antioxidant HO-1 may directly interact with ROS in the presence of metal ions, such as copper and iron, ultimately leading to cell death through the ferroptosis pathway [134]. Carotenoids have been shown to inhibit GPX4, promote ROS accumulation and lipid peroxidation, and induce ferroptosis in PCa cells [135]. These studies suggest that increasing ROS levels in PCa cells may offer a promising therapeutic strategy by promoting prostate cell death. However, as noted above, ROS can also promote the proliferation of PCa cells. Therefore, balancing ROS levels to avoid their dual role in promoting prostate cancer progression remains an area for further investigation.

## Therapeutic strategies: modulating oxidative stress in prostate cancer management

PCa treatment strategies vary depending on the stage of the disease, with ADT remaining the primary approach. For patients with metastatic CRPC, the standard treatment involves a combination of ADT, AR inhibitors, and chemotherapy. Study has shown that androgens can induce the production of ROS in prostate cancer cells. This ROS production triggers a preemptive cellular response to counteract oxidative stress, allowing prostate cancer cells to adapt to radiation and thereby reducing the efficacy of radiotherapy [136]. Further research has indicated that androgen-induced ROS production is mediated through the upregulation of NOX2 and NOX4 expression [137]. Blocking this ROS upregulation using apocynin or androgen receptor antagonists enhances the sensitivity of prostate cancer cells to radiotherapy [137]. These findings suggest that inhibiting ROS production in prostate cancer cells may have a beneficial effect in improving the response to radiotherapy. Given that oxidative stress contributes to the proliferation and invasion of PCa and its progression to CRPC, the use of antioxidants to eliminate or prevent ROS generation presents a potential therapeutic strategy for managing PCa. As shown in Table 1, studies have demonstrated the efficacy of antioxidants in the chemoprevention of PCa.

**Table 1:** Antioxidants and their therapeutic implications in prostate cancer

Antioxidants	Effect/Mechanism	References
Zinc	Dietary zinc intake is associated with lower prostate cancer-specific mortality, especially in men with localized disease	[138]
SY	Reduce biomarkers of oxidative stress	[140]
Calcitriol	Lower PSA levels and enhance patient survival rates	[141]
Tea	Reduce the risk of prostate cancer	[144]
EGCG	Reduce circulating testosterone levels and inhibit prostate growth	[145]
Tangeretin	Inhibit the proliferation of PCa cells by suppressing the PI3K/Akt/mTOR pathway	[148]
Proanthocyanidins	Reduced the risk of prostate cancer, especially in cases with a Gleason score ≥ 7	[150]
MPX	Increase PSADT by 5.3 months in BRPC patients	[151]
flavanol dimers B1-B4	Reduce prostate cancer growth by targeting mARs	[152]
genistein	Improve the inhibition of primary tumor growth (87%) compared with genistein (30%) or radiation (73%) alone	[149]

*SY* selenium-enriched yeast, *MPX* Pulverized muscadine grape, *BRPC* Biochemically recurrent prostate cancer, *PSADT* Prostate-specific antigen doubling time, *mARs* Membrane androgen receptors

Zinc, a trace element that serves as a cofactor for SOD, is crucial for the enzyme's antioxidant activity. Research by Epstein et al. [138] indicated that increased dietary intake of zinc is associated with a reduced prostate cancer-specific mortality rate. Similarly, selenium, another trace element, has shown promise in PCa treatment. However, the form and dosage of selenium can affect its efficacy, with yeast-derived selenium exhibiting stronger antioxidant effects compared to selenomethionine [139, 140]. Common antioxidants, such as vitamins D and E, have also been studied for their potential benefits. A double-blind trial demonstrated that calcitriol, the active form of vitamin D, can lower PSA levels and extend survival in PCa patients [141]. Vitamin E has also been shown in clinical trials to reduce PCa risk [142]. However, the small sample size in these studies has led to inconsistent findings, with larger clinical trials indicating that neither vitamin E nor selenium, whether used alone or in combination, effectively reduces PCa risk and may even increase it [143].

Natural antioxidants have been found to have a beneficial role in PCa treatment. Although the association is not strong, some studies suggest that regular tea consumption, which is rich in antioxidants, may reduce the risk of PCa [144]. The primary antioxidant in tea is epigallocatechin gallate (EGCG), which has been shown to significantly reduce obesity and circulating testosterone levels, thereby inhibiting prostate growth [145]. Gann's study found a significant association between plasma lycopene levels and reduced PCa risk, with higher lycopene concentrations correlating with a lower risk of PCa, particularly in more aggressive forms of the disease [146]. However, another study found no association between lycopene and PCa risk [147], indicating that the effectiveness of lycopene in preventing or treating PCa requires further investigation. Flavonoids, a class of plant-derived compounds, have emerged as natural antioxidants with potential therapeutic benefits in PCa. Zhu et al. [148] reported that tangeretin, a flavonoid derived from citrus, can inhibit the growth of androgen-insensitive PC-3 cells and androgen-sensitive LNCaP cells by suppressing the PI3K/Akt/mTOR pathway. Additionally, the flavonoid genistein has been shown to significantly enhance the effectiveness of radiotherapy in PCa treatment. When used in combination, genistein and radiotherapy achieved an 87% tumor inhibition rate, compared to 30% with genistein alone or 73% with radiotherapy alone [149]. Proanthocyanidins, a group of natural polyphenolic compounds, have also been found to be inversely associated with PCa risk, with the association being particularly strong in patients with a Gleason score of 7 or higher [150]. Resveratrol, a polyphenol found in MPX, has demonstrated anti-PCa activity in clinical settings, with MPX use prolonging PSA doubling time in patients [151]. Anthocyanins, another class of antioxidants, can scavenge ROS, and studies have shown that oligomeric proanthocyanidin B1-B4 dimers can reduce the growth of LnCaP and DU145 cells, with B2 showing the strongest anti-proliferative effects [152].

## Conclusions

The excessive generation of ROS and the resulting increase in oxidative stress play a pivotal role in the progression of PCa. In PCa cells, the antioxidant systems, including SOD, CAT, GPX, and zinc ions, are impaired to varying degrees. Furthermore, elevated androgen levels can further disrupt the ETC, leading to increased ROS production. External factors such as aging and obesity also contribute to the rise in ROS levels. The combined damage to the antioxidant system and the increased ROS production result in heightened oxidative stress, which in turn promotes PCa progression and metastasis by enhancing angiogenesis, altering the extracellular matrix, promoting inflammation, activating the UPR, and regulating miRNA expression. Given the role of oxidative stress in promoting PCa, antioxidants have been explored as a therapeutic strategy. However, clinical trial data on antioxidant therapy in PCa are still inconclusive and require further validation, particularly regarding the use of natural products in PCa treatment. Moreover, considering the positive role of ROS in the progression of CRPC, future research should focus on the relationship between ROS and CRPC, with the aim of advancing antioxidant strategies for CRPC management.

## Data Availability

No datasets were generated or analysed during the current study.
